# Practical Bayesian Inference in Neuroscience: Or How I Learned To Stop Worrying and Embrace the Distribution

**DOI:** 10.1101/2023.11.19.567743

**Published:** 2023-11-21

**Authors:** Brandon S Coventry, Edward L Bartlett

**Affiliations:** 1 Department of Neurological Surgery and the Wisconsin Institute for Translational Neuroengineering, University of Wisconsin-Madison, Madison, WI USA 53705; 2 Weldon School of Biomedical Engineering, Department of Biological Sciences, and the Institute for Integrative Neuroscience, Purdue University, West Lafayette, IN USA 47907

**Keywords:** Bayesian Inference, Neural Data Analysis, Statistical Inference

## Abstract

Typical statistical practices in biological sciences have been increasingly called into question due to difficulties in replication of an increasing number of studies, much of which is confounded by the relative difficulty of null significance hypothesis testing designs and interpretation of p-values. Bayesian inference, representing a fundamentally different approach to hypothesis testing, is receiving renewed interest as a potential alternative or complement to traditional null significance hypothesis testing due to its ease of interpretation and explicit declarations of prior assumptions. Bayesian models are more mathematically complex than equivalent frequentist approaches, which have historically limited applications to simplified analysis cases. However, the advent of probability distribution sampling tools with exponential increases in computational power now allows for quick and robust inference under any distribution of data. Here we present a practical tutorial on the use of Bayesian inference in the context of neuroscientific studies. We first start with an intuitive discussion of Bayes’ rule and inference followed by the formulation of Bayesian-based regression and ANOVA models using data from a variety of neuroscientific studies. We show how Bayesian inference leads to easily interpretable analysis of data while providing an open-source toolbox to facilitate the use of Bayesian tools.

## Introduction

Inference tools are foundational to all studies in neuroscience, providing the necessary machinery to make decisions and conclusions from data. Frequentist-based null significance hypothesis testing (NHST) has been the gold standard of inference in neuroscience and science at large in part due to the computational simplicity of frequentist models compared to permutation sampling or Bayesian-based methods. A significant problem present in the current practice of NHST, however, arises in the adoption of the p-value as the *de facto* metric of experimental “success”, notorious for its difficulty in interpretation and correct usage (Krueger and Heck, 2019). The confluence of exponential increases in computational power with the wider discussion of problems with NHST usage has created renewed interest in Bayesian inference as an alternative to frequentist NHST while offering interpretability benefits over the p-value and NHST overall.

The use of p-values, the ubiquitous decision rule in frequentist methods, is fraught with problems due to fundamental misunderstandings of its use, interpretability, and most pathologically, its susceptibility to intentional and unintentional p-hacking(Nuzzo, 2014). Contrary to the initial intent of Ronald Fisher(Fisher, 1992), the p-value has often become the gatekeeper of significance in studies. In this role, it limits deeper observations into data, and it is often used without proper experimental design to ensure proper use and control. Methods of statistical inference require that one first define a statistical model with the power to adequately describe the data-generating process. Inference is then performed to estimate the population distribution from limited samples of observed data. Once estimates of population distributions are made, the determination of whether or not these distributions represent a significant effect is determined. NHST is somewhat a victim of its own success, where common practice has distilled the practice of NHST to chase the somewhat arbitrary p<0.05 measure of significance devoid of model or data considerations(Krueger and Heck, 2019). Furthermore, even in the best of experimental designs, the p-value is a surrogate for arguably what a researcher is most interested in: how likely is it that observed data has some effect different from null(Kruschke, 2011; Gelman and Shalizi, 2013).

Bayesian methods offer a solution to the problem of the p-value, providing a direct measure of the probability that observations have some effect(Kruschke, 2011; Gelman and Shalizi, 2013). This is done by reallocation of probability of possibilities as parameters in a mathematical model of the data-generating process, leading to probabilistic estimates desired by but not attainable with p-value analyses. Bayesian methods are inherently data-driven; models are built with prior knowledge directly incorporated from parameters estimated directly from observed data.

Bayesian inference, though chronologically younger than frequentist approaches, was not adopted as the primary inference paradigm due to the computational demands necessary to solve inference problems outside of certain canonical forms(Bishop, 2006) and the adoption of frequentist interpretation of probability(Fienberg, 2006). Inference on arbitrary distributions required a deeper mathematical knowledge and computation of integrals which were potentially intractable without modern numerical integration techniques. Frequentist paradigms however were more easily adapted to computationally simple algorithms, allowing researchers to “do statistics” without extensive formal training. However, exponential increases in computational power with the development of powerful Markov chain Monte Carlo (MCMC) sampling methods now allow researchers to perform meaningful Bayesian inference on arbitrary distributions underlying observed data(Gilks et al., 1996).

The goal of this tutorial is to remedy the opacity that often accompanies discussions of Bayesian inference by providing simple, step-by-step walkthroughs of Bayesian inference with four common inference paradigms. We also aim to demonstrate the explanatory power of Bayesian inference in the context of neuroscience data. While the aim of this article is focused on application, this tutorial will begin with a brief introduction to Bayes’ rule and its constituent components necessary for inference. For more theoretical and mathematical considerations of Bayesian inference, see the following books and articles(Gerwinn et al., 2010; Colombo and Seriès, 2012; Bielza and Larranaga, 2014; Kruschke, 2014; Kruschke and Vanpaemel, 2015; Ma, 2019; Gelman et al., 2021; Van De Schoot et al., 2021).

## Estimation of Spike Rates from Auditory Stimuli: A Motivating Example

**Figure 1:**
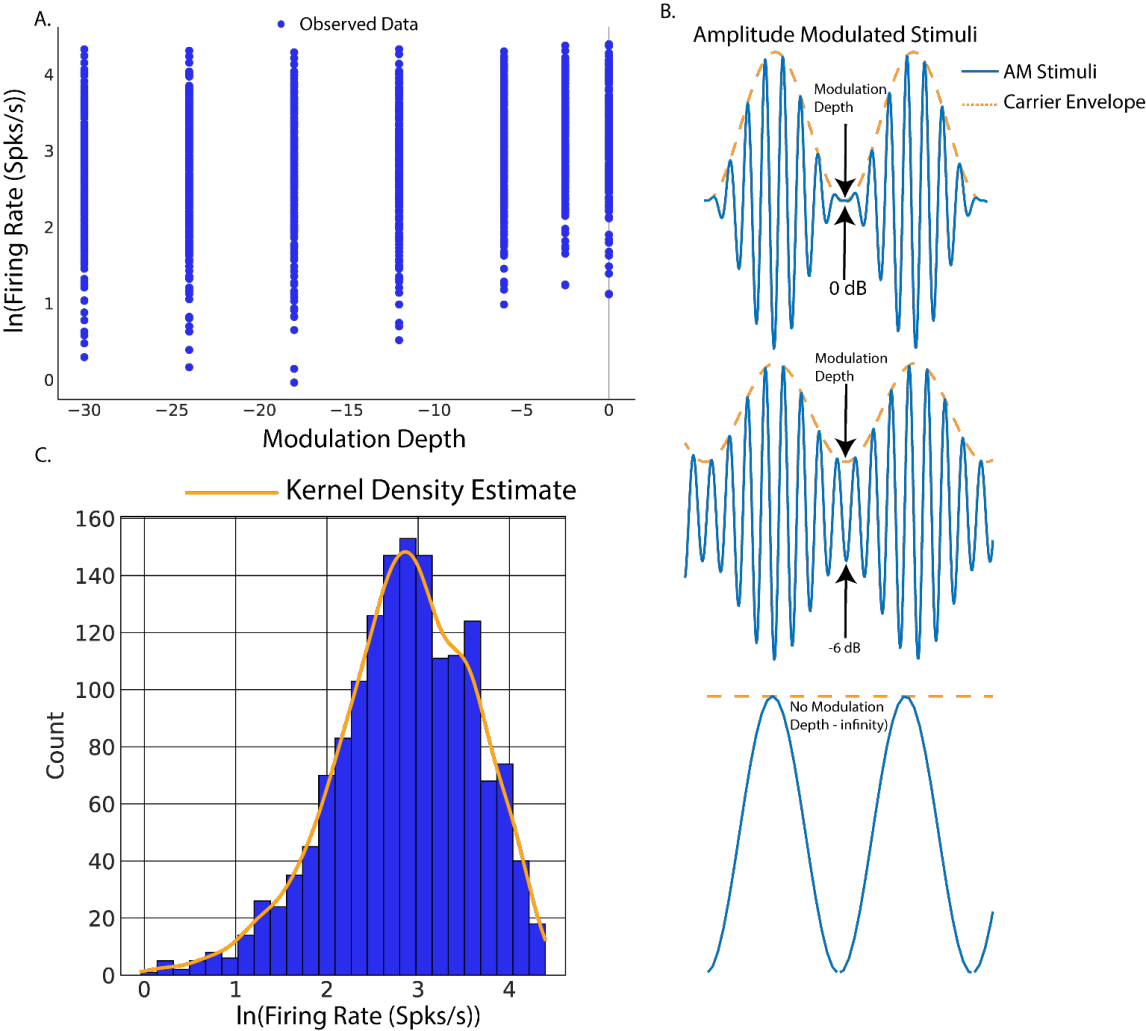
Example of Bayesian simple linear regression on population estimates of firing rate vs amplitude modulation depth stimuli. This model was applied to population single unit firing rates elicited from inferior colliculus with sinusoidal amplitude modulated (SAM) tones. The goal of this model was to predict evoked firing rates from increases in SAM modulation depths. A. Scatterplot of observed firing rates vs SAM modulation depth and fitted regression estimates. B. Schematic of amplitude modulated stimuli. C. Kernel density estimates of the observed log transformed data probability distribution function. C. An example of Bayesian model comparison. Left: Regression model with untransformed data. Right: natural log transformed firing rate model. Posterior predictive checks reveal that natural log transformed firing rate models better match observed data.

To facilitate the discussion of Bayesian inference in neuroscience, consider an example found prominently in auditory neuroscience(Fig 1A–B). In our first experiment, single unit recordings were made from the inferior colliculus (IC) in response to applied sinusoidal amplitude-modulated tones (SAM, see SI Methods). The goal of this analysis is to create a linear model of SAM temporal auditory processing by quantifying increases in evoked single unit firing rates in response to decreased SAM modulation.

The linear regression model seeks to estimate a linear relationship between one (simple linear) or more (multilinear) predictor and measured variables. In this model, both the measured result and predictors are metric variables which map to a continuum of possible values. The simple linear regression model takes the form of:

y=α+βx+ϵ

where *y* is the measured (predicted) group, *x* is the predictor, *β* is the “slope” parameter dictating the relative increase or decrease in *y* per unit change in *x*, *α* is the intercept term which, in models of firing rate represents non-evoked, spontaneous firing rates, and *ϵ* is an error term which quantifies the difference between the expected value of *y* at a given *x* given a linear model versus the observed value of *y* at *x*. It should be noted that *ϵ* is not present in all regression models, but the authors suggest inclusion to quantify deviations from linear fit.

Linear regression thus forms a model in which AM depth predicts evoked firing rates in which the model parameters are estimated and used to draw conclusions about the relative dependency of *y* on *x*. To begin, an observation of the relative distribution of the measured data, in this case firing rates elicited from IC, will allow for robust inference model design. Inspection of the distribution of firing rates (Fig 1C) suggests that a log transform would allow for the data to be normally distributed, making model computations easier through use of canonical normal distributions. Before continuing to inference, it is important to describe the functional components of Bayesian inference’s computational tool; Bayes rule.

## Bayes’ Rule

**Figure 2:**
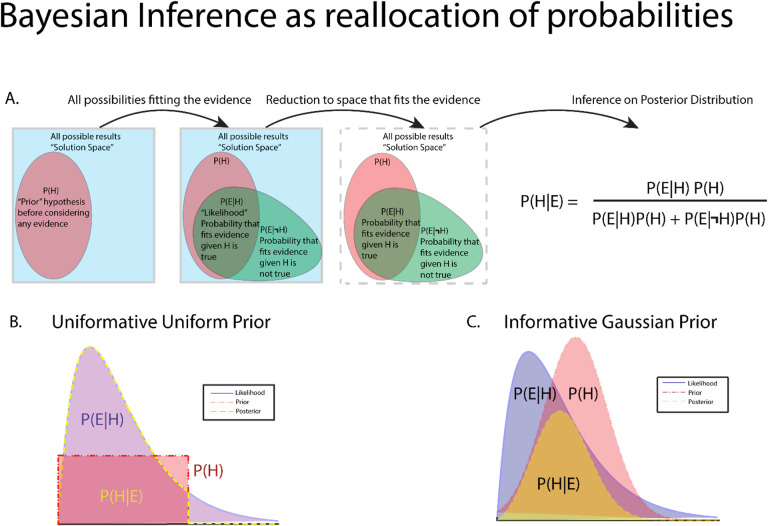
Graphical description of Bayes rule and the interaction between prior distributions and likelihood functions leading to the final posterior distribution. A. Bayes rule can be thought of as a reallocation of probability to the posterior after accounting for prior distributions and observed evidence. B. An example of posterior generated from an inverse-Gamma distributed likelihood and a uniformly distributed prior. Uniform priors reflect the likelihood function, and thus the observed data with no redistribution probability, making uniform distributions uninformative priors. However, care must be taken in using uniform distributions as observed data outside of prior bounds is mapped to 0 probability. C. An example of a posterior generated from an inverse-Gamma distributed likelihood and a gaussian distributed prior. This prior is considered informative as it shapes the posterior distribution to a greater extent than a uniform distribution. Prior distributions with longer tails can handle extremes of observed data by mapping extreme events to low, but non-zero representation in the posterior. Examples B and C represent extremes of prior choices, with minimally informative priors often chosen to let the data “speak for itself” with little change to posterior from prior influence.

Foundational to Bayesian approaches is a complementary, but epistemically differing view of probability from that of frequentist approaches. While the frequentist perspective treats probability as the **relative frequency** of the occurrence of some event, the Bayesian perspective instead treats probability as the **expectation** of an event occurring which can be used to not only quantify the state of knowledge of an event, but also the uncertainty involved in measuring an event. Traditionally, the Bayesian perspective has been called ‘belief’, a perhaps unfortunate name which belies the fact that the Bayesian perspective of uncertainty of an event is fundamentally quantifiable. Perhaps a better description of Bayesian belief is instead quantification of the state of knowledge by accounting for uncertainty. The cornerstone of Bayesian inference is Bayes rule, defined as:

$$P(H|E)=\frac{P(E|H)P(H)}{P(E)}$$

where H is the quantification of the state of a hypothesis, and E is the quantification of observed evidence. In the context of inference, it is helpful to explicitly state the role of the model in Bayesian formulations:

$$P(\theta |E,M)=\frac{P(E|\theta ,M)P(\theta |M)}{P(E|M)}$$

where M is the model of the data generating process and *θ* are the model parameters. The individual components of Bayes’ rule are given names corresponding to the purpose they serve, with *P*(*θ*|*E*, *M*) called the posterior distribution, *P*(*E*|*θ*, *M*) the likelihood function, *P*(*θ*|*M*) the prior distribution, and *P*(*E*|*M*) the evidence or marginal likelihood function. Taken together, Bayes’ equation represents the quantification of observed data accounting for prior knowledge(Fig 2A). Each component plays a key role in Bayesian inference and each will be discussed briefly below.

### The Model Evidence

The denominator term *P*(*E*|*M*), called the model evidence (or just the evidence or marginal likelihood in Bayesian parlance) is the quantification of the probability of observing the data under a chosen model of the data generating function. At first glance, the calculation of the total evidence appears to be an insurmountable task. In reality this term is the weighted average of parameter values in a given model weighted by the relative probability of a given parameter value(Kruschke, 2014) and thus acts as a normalization term to ensure the numerator is a proper probability distribution. The structure of *P*(*E*|*M*) will change based on whether the distributions represent probability mass functions (discrete case) or probability density functions (continuous case). In the discrete case, the evidence is

$$P(E|M)=\sum_{\theta }p(E|\theta ,M)p(\theta |M)$$

and in the continuous case:

P(E∣M)=∫p(E∣θ,M)p(θ∣M)dθ

The evidence function thus represents an average of the likelihood function across all parameter values conditioned on the prior distribution. The marginal likelihood can also be utilized to assess the plausibility of two competing models(Johnson et al., 2023). The evidence, especially in the continuous case, is historically what made Bayesian inference difficult due to the need to evaluate a complex integral numerically. However, the advent of Markov-chain Monte Carlo (MCMC) methods with improvements in personal computer processing power has allowed for computationally efficient integration without the need for supercomputing hardware. MCMC methods will be discussed in a subsequent section.

### The Prior

The prior, *P*(*θ*|*M*), is often the major stumbling block for those entering into Bayesian inference, but this hurdle is less about the prior, and more about what the prior is perceived as. The prior, *P*(*H*) describes the investigators prior beliefs on the state of knowledge of the study. Critics of Bayesian inference have described the prior as purely subjective, but we, and many others(Kruschke, 2010; Box and Tiao, 2011; Gelman and Shalizi, 2013), argue that the prior represents an explicit declaration of the investigators knowledge, assumptions, and the general state of a field which is implicit and often is present but not stated in frequentist approaches. Moreover, one is encouraged to perform prior predictive checks to compare the sensitivity of competing priors in a Bayesian inference model, as we will show subsequently. The practice of the design of experiments and their resulting publications are rife with implicit priors which are often not acknowledged or realized when reporting results. As an example, consider study of cortical extracellular single unit recordings(Paninski et al., 2004; Bartlett and Wang, 2007; De La Rocha et al., 2007; Coventry et al., 2023a as illustrative examples). The investigator could be leading a project with vast knowledge accumulated over years of study. Or the investigator is a trainee of a career researcher who draws a view of cortical physiology from their experienced mentor mixed with reading current literature. When designing an experiment, the investigator will have some intuition regarding likely and biologically feasible resting state and stimulus-evoked firing rates, cognitively assigning relatively low likelihood of seeing extremes of firing rates with higher likelihood assigned to moderate firing rates previously observed in literature or seen in experiments, and likely will discard or treat as outliers firing rates on the extremes or thought to be non-biological noise. The power of the prior distribution in Bayesian approaches is in part the need to explicitly quantify and report these prior beliefs, which can be analyzed and scrutinized as part of the peer review or post-publication process. Prior distributions also require investigators to consider their biases and relative expectation on the importance of previously recorded and read data, promoting a deeper understanding of not only the data obtained within their lab, but also of the general state of the specific neuroscience field. As the name implies, prior beliefs are quantified as probability distributions by the investigators.

This begs the question as to what a prior might look like in newer avenues of study where a paucity of data exists. Or in situations where researchers and data analysts want the data to “speak for itself” outside any influence of the prior. In these cases, priors can be designed to be “non-informative” or “weakly-informative”, assigning broad, non-committal distributions to the prior. One might assign a uniform distribution on the prior, effectively treating each parameter outcome as equally likely. Uniformly distributed priors do require some caution, however, as any parameter value outside of the bounds of the uniform distribution is automatically assigned probability 0 in the posterior, even if that value has been observed(Fig 2B). In many cases, it’s better to allow small, but nonzero probabilities to extreme values, such as the tails of a normal distribution, such that evidence for unexpected events is represented in the posterior given strong data(Fig 2C). Conversely, priors can be made to be highly informative in situations where physiological bounds are well known and well-studied, where extreme values are known to be biophysically irrelevant or impossible or known to be due to instrument noise(e.g. large 50/60 Hz noise peak in power spectrum indicative of wall power noise).

### The Likelihood

The likelihood function, *P*(*E*|*θ*, *M*) describes the probability that data is observed given parameter values *θ* in a data generating model M. In the context of inference, the likelihood function updates information given in a prior distribution to the posterior distribution given the observed data(Etz, 2018). The likelihood function is generally not a proper distribution, in that it is conditioned on yet unknown parameters and may not integrate to 1, but the evidence and prior terms ensures that resultant posterior distributions are true probability densities. The idea of likelihood functions are present in both Bayesian and frequentist models, but has vastly different interpretations. The model parameters in a frequentist viewpoint converge upon singular values learned, usually though maximum likelihood estimation, from merging competing hypotheses of data. Bayesian approaches treat model parameters as ranges arising from distributions after observing the data at-hand.

### The Posterior

The prior, likelihood, and evidence then form the posterior *P*(*θ*|*E*, *M*), the reallocation or mapping of probability from likelihood function, prior, and model evidence to an all-encompassing distribution. The posterior thus is the evidence for parameters *θ* conditioned on observed data and a model of the data generating function. The posterior forms the basis for inference, with all relevant information encoded in its distribution. Inference on the posterior distribution is covered in a section below.

### Estimation of the Posterior

Despite Bayes’ rule being formulated before Fisher’s description of frequentist methods, a major reason that Bayesian inference was not been widely adopted was fundamentally a computational one, in that evaluation of Bayes’ rule often requires solving non-trivial integrals. A subset of computationally tractable prior distributions and likelihood functions formed canonical posteriors in which the posterior is easily inferred. However, these cases are not generalizable to experimental data which can be noisy and not well behaved. Modern Markov-chain Monte-Carlo (MCMC) tools have been developed to quickly and easily estimate arbitrary distributions. MCMC involves the generation of random samples which converge to a target probability distribution, the details of which can be learned from the following reviews(Hoffman and Gelman, 2011; Betancourt, 2017).

### Making Decisions on the Posterior

We define inference broadly as the process by which reasoning and decisions about a phenomena are made from a sample of observations of the phenomena. Classical NHST does not offer zero probability of error hypothesis testing(Blackwell, 1980). However, incorporation of prior knowledge in Bayesian inference allows for optimal decision making on observed data(Blackwell and Ramamoorthi, 1982). The posterior contains all necessary information to make inferences on experimental data incorporating prior knowledge. However, it is best to consider the specific goals of inference before performing statistics. Possible goals of inference are as follows(Kruschke, 2014):

- Infer the parameters of a model.
- Reject or confirm a null hypothesis
- Compare two or more competing models

In the case of neuroscientific studies, inferring model parameters occurs when an experiment aims to establish how neural firing rates change with changes in applied stimuli. Or one may want to confirm and reject a null hypothesis that a treatment has the desired effect or that there are differences between neural populations. Importantly, because the Bayesian inference operates solely on the posterior distribution, one can confirm or reject competing hypotheses and not simply reject the null as in frequentist NHST.

Regardless of the goal, inference always involves analyzing the posterior, which provides a complete representation of the distribution of a given parameter given the experimental data. Therefore, decisions about the data, the effect of model parameters, and/or which hypothesis has more evidence is performed with calculations on the posterior. There are a multiplicity of decision rules that can be used to assess the posterior. The most common, and in the author’s opinion, the most intuitive is that of the Bayesian credible interval. The confidence interval calculates the probability that a population parameter lies in a certain interval. As credible intervals are not strictly unique, Bayesian inference convention is to fix the interval to the smallest interval which contains 95% of the posterior distribution density mass called the highest density interval (HDI). Observations of posterior HDIs can then be used to assess the relative effect of a parameter. Regions of practical equivalence (ROPE) may be included in the posterior distribution that explicitly define a range of values that are effectively equivalent to a null value, with parameters considered significant if 95% of the posterior distribution (95% HDI) does not contain 0 or any values in the ROPE (Kruschke, 2018). Along with posterior HDIs, calculations of maximum *a posteriori* (MAP, distribution mode) estimates from the posterior are performed to quantify a most likely parameter value. While decision rules are important to assess the relative effect of statistical model parameters, we reiterate that simply passing a decision rule should not conclude the inference step. Inference should be made in context of the evidence presented in model quality checks, observed data posterior distributions, and decision metrics.

### Error Quantification and Model Comparison

Critical to any statistical model and inference therein is its fit to observed data. While it is entirely possible to perform linear regression on data distributions which are highly nonlinear, predictions and inference made by the model will likely be inaccurate. Both Bayesian and frequentist inference offer robust model error quantification. Bayesian approaches, however, can utilize the posterior distribution to not only quantify and bound the distribution of model errors, but also include *post hoc* posterior predictive sampling as part of the inference paradigm. Posterior predictive sampling involves making random draws from the posterior and building a sampling distribution. This distribution is then compared to the observed data distribution to quantify the model’s disparity from observed data. Along with posterior predictive checks, prior predictive checks act as a sensitivity measure of the influence of the prior distribution on the posterior distribution. Taken together, Bayesian inference thus allows for robust statistical inference on observed experimental data which appropriately includes prior knowledge of the state of the field.

### Formulation of Models and Applied Bayesian Inference

There are a multiplicity of programs and programming languages that facilitate Bayesian analysis, such as standalone programs of Jasp(Love et al., 2019) and probabilistic programming language packages such as BUGS(Brooks, 2003) and STAN(Carpenter et al., 2017), we chose to use PyMC(Salvatier et al., 2016) for its ease in explicitly declaring probability distributions and its implementation in Python which is in common use in neuroscientific data analysis. Model formation is often conserved between frequentist and Bayesian approaches; it is only the mode of inference that differs. However, for clarity, we will discuss both model formation and performing inference in the subsequent sections.

## Performing Bayesian Inference on the Linear Regression Model

Turning back to the example of IC single unit firing rates in response to SAM depth stimuli, the first step in inference is to place a prior distribution on the data. Previous studies and data can be used to inform the prior, but for this example we chose to demonstrate regression with moderately informative priors on *α*, *β*, and *ϵ* so as to let observed data drive posterior inference. Given that the data observed data is roughly normal, a good first pass is to place a normal distribution on the prior with mean equal to the mean of the observed data and a variance that is wide enough to capture all observed data. After inference is made, sensitivity analyses can be performed to assess the relative importance of the prior parameter values on posterior estimates. Larger prior variances allow for small, but non-zero probabilities on extreme values. This tends to be a more robust approach than setting a value of 0 on extreme events, as observed data with strong evidence for an extreme value can be adequately represented in the posterior. After observation of the underlying distribution of the observed data and decision on a prior distribution, a linear regression inference model can be easily described in code as follows:

Code Example 1: PyMC initialization of a simple linear regression model


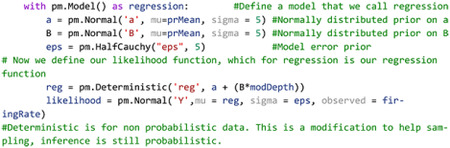


The likelihood variable then translates our model to one of Bayesian inference by casting the model as a probability distribution, in this case

y~N(α+βx+ϵ)

noting that observed firing rates are incorporated by the ‘observed’ parameter in the likelihood distribution. To generate the posterior, all that needs to be done is to initialize and run MCMC as follows:

Code Example 2: Running the MCMC sampler


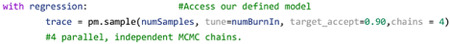


This routine then generates a trace variable containing the posterior distributions of all model parameters after sampling numSamples with numBurnIn samples to initialize chains. We also ran 4 chains in parallel with a target_accept probability of 90%. Acceptance probability is somewhat based on the statistics of observed data and model, with more difficult posteriors benefiting from higher accept probability values(Gilks et al., 1996). Improper acceptance probabilities can give rise to insufficient number of draws and malformation of posterior distributions. PyMC provides a helpful readout for when posterior draws are malignant and indicative of higher acceptance probabilities. In summary, in a few lines of code the researcher has observed distributions of the data and explicitly defined a model of the data generator and likely now has a better intuition of the data and how it is distributed. All that’s left to observe the posteriors with HDIs to infer significance from the model.

**Figure 3:**
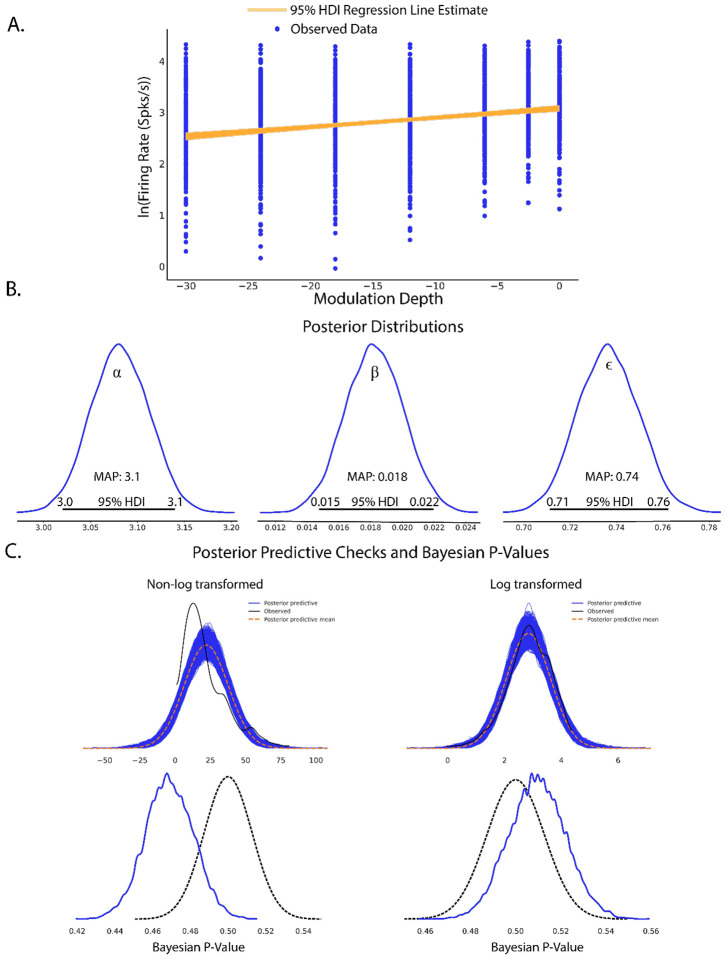
Completed Bayesian inference quantifying linear relationships in evoked firing rate from increases in modulation depth. A. Scatterplot of observed firing rates vs SAM depth stimuli with fitted regression line estimates superimposed. 95% HDI estimates of regression slopes are shown in orange, with the spread of lines encoding the 95^th^ percentile of most likely slope values. B. Estimates of Bayesian linear regression parameters. Intercept term *α* was significantly above 0 (MAP = 3.1, 95%HDI does not overlap 0) which indicates basal firing rates above 0. Regression slope was small but significantly above 0 (MAP = 0.018, 95% HDI does not overlap 0) suggesting an increase in evoked firing rates with increased modulation depth. Error term *ϵ* was significantly above 0 (MAP = 0.74, 95% HDI does not overlap 0) suggesting some model deviation from observed data. However, error terms were considered small as *ϵ* MAP < *α* basal firing rate MAPs. C. Posterior predictive checks of linear (left) and log linear (right) regression models show that log transformed firing rate models produce posterior predictions most inline with observed data. Disparity of empirical posterior predictive distributions from observed data as quantified through Bayesian P-values also suggest log transformed firing rates creates a superior model fit.

Plotting the 95% HDI estimation of the regression line (Fig 3a) on modulation depth vs natural log-transformed firing rates suggest a small but significant increase in firing rates with increases in modulation depth. Posterior distributions of model parameters (Fig. 3B) also show that there is an estimated basal firing rate above 0 (α MAP = 3.1) and a slope increase small but significantly above 0 (β MAP = 0.018) with model error terms considered small for being significantly smaller than intercept term (ε MAP = 0.74). The spread of the 95% HDI on inferred parameters is used as a measure of uncertainty of the parameter, with narrow HDIs representing more certainty in MAP estimated parameter. In our model, the *α* parameter has a spread between 3.02 to 3.13, with a difference of 0.11 containing 95% of its posterior distribution, suggesting strong certainty in the MAP estimate of 3.1. Similar narrow spread is see in the *β* parameter, with a difference of 0.007 containing 95% of the posterior. The model error term shows that observed data deviation from the model is constrained between 0.71 and 0.76 suggesting relative certainty in the magnitude of deviation of the data from the model.

Statistical conclusions should not end after making inferences on model parameters however. Critical to the validity of statistical inference is the quality of the model fit to observed data. This goodness of fit in Bayesian approaches can be analyzed by posterior predictive checks, in which sample draws are made from the posterior distribution, simulating observations of data generated from the experiment from which the statistical model was fit, and comparing sampled to observed data to assess deviation of model predictions from observed data distributions. In PyMC, posterior predictive checks can be easily performed using the following code:

Code Example 3: Performing posterior predictive checks


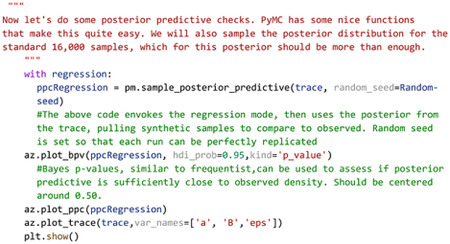


To illustrate how posterior predictive checks can be used, a competing model was made which performs Bayesian linear regression to the same data and priors except without log transformation of the data. In each case, random draws were made from each log transformed and non-log transformed posteriors to create empirical data distributions. Comparison of empirical distributions qualitatively show that log-transformed models present a better fit to observed data than non-log transformed models. The relative disparity between posterior predictive model fits and observed data can be quantified by use of Bayesian p-values, a distance measure between two distributions( for details of Bayesian p-values, see Kruschke, 2014). The closer the Bayesian p-value is to 0.5, the better data sampled from the posterior overlaps with the distribution of observed data. Plotting the resulting distributions and the Bayesian p-values indeed show the log-transformed model fits better to observed data than the non-transformed model. Similar analyses can be performed around model free parameters, such as prior variables, to form a sensitivity analysis of prior distributions on resulting posterior inferences.

A secondary and quick check of posterior sampling can be performed by qualitative evaluation of the MCMC sampling chains, often called traces. Traces represent the long term run of a Markov chain which represent the distribution of interest. As such, good traces show evidence of effective sampling and convergence to target probability distributions. PyMC offers easy ways to visualize posterior MCMC traces using the *plot_trace* function. Figure 3 shows traces obtained from our Bayesian regression example. Kernel density estimates of traces corresponding to the posterior distributions of regression parameters show good convergence of MCMC traces to a target distribution (Fig 3A). As MCMC chains are time series samples which form a distribution, evaluation of traces through sampling time can also be used as a diagnostic of sampling convergence. Traces should have a “fuzzy caterpillar” like appearance (Fig 3B) without any stark jump discontinuities from sample to sample. Quantitative trace evaluations are also available, with the Gelman-Rubin statistic ($\hat{r}$) being the most prominent. The Gelman-Rubin statistic measures the variance between MCMC chains to the within chain variance, effectively measuring chain stationarity and convergence(Gelman and Rubin, 1992). Heuristically, $\hat{r}<1.05$ is considered good convergence of MCMC chains. This value can be calculated *post hoc* after sampling and PyMC will automatically flag if $\hat{r}\geq 1.05$ is detected.

While there are many reporting guidelines for Bayesian inference, we follow the Bayesian Analysis Reporting Guidelines as given by Kruscke(Kruschke, 2021) and provide an example reporting document including posterior predictive checks, Bayesian model comparisons, and sensitivity analysis as supplementary material.

### Multilinear Regressions, Repeated Measures, and Hierarchical Models

In many experiments, inference across multiple possible data generating parameters must be analyzed and accounted for. These models, called multilinear regressions, are extensions of standard linear regression as follows:

$$y=X^{T}\beta +\epsilon \to y=\beta_{0}+\beta_{1}x_{1}+\beta_{2}x_{2}\ldots +\beta_{n}x_{n}+\epsilon$$

where n is the total number predictors.

**Figure5:**
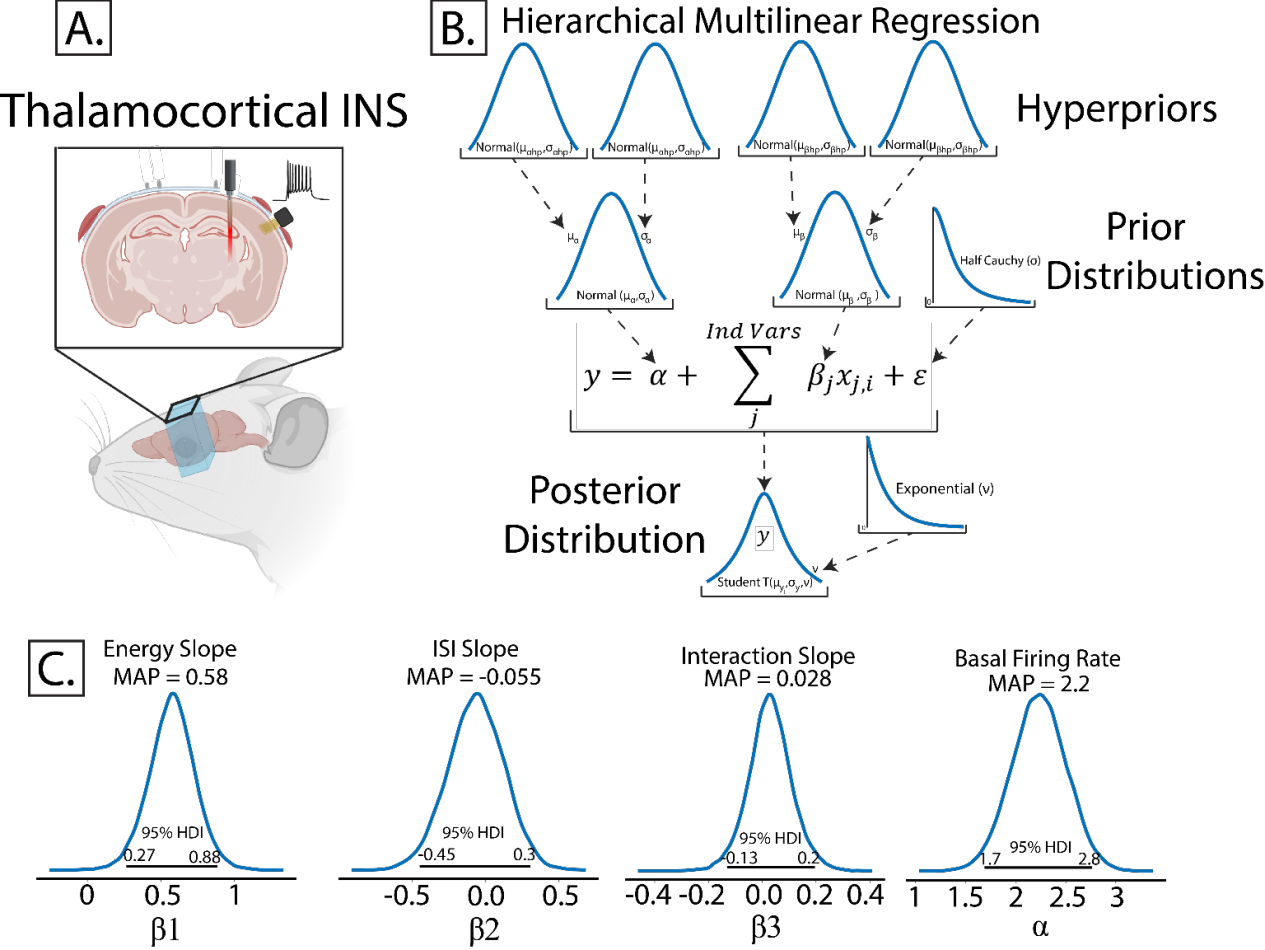
Example of Bayesian multilinear regression incorporating a hierarchical structure. A. In this experiment, rodents were implanted with fiber optic arrays into auditory thalamus and planar recording arrays into auditory cortex. Single unit responses were recorded from INS stimuli with applied energy and interstimulus intervals varied to derive dose-response curves. Figure was drawn using BioRender under publication license (www.biorender.com). B. Hierarchical schematic of Bayesian multilinear regression. Hierarchical structures are advantageous in accounting for within and between subject variability or for repeated measures designs. C. Resulting parameter distributions from dose-response models. Energy was a significant contributor to maximum firing rate, with increasing laser energy resulting in increased maximum firing rate, as determined by 95% HDI of the laser energy term *β*_1_ excluding 0 (MAP = 0.58). Laser pulse interstimulus interval did not significantly contribute to changes in max firing rate as indicated by ISI parameter *β*_2_ overlapping 0 in its 95% HDI with a MAP value near 0 (MAP = 0.028). The relatively wide spread about zero does suggest that there may be a subset of ISIs which contribute more strongly to firing rates and warrants further study. Laser energy-ISI interactions also did not significantly contribute to max firing rate as evidenced by interaction parameter *β*_3_ including 0 in its 95% HDI. The intercept term *α*, correspondint to basal firing rates, were significantly above 0 (MAP = 2.2, 95% HDI excludes 0).

To illustrate the use of multilinear regressions, consider the case of thalamocortical infrared neural stimulation (INS)(Fig 5A). Auditory thalamic neurons in the medial geniculate body were excited by pulse trains of optical stimuli varying in pulse energy and time between pulses. The resulting auditory cortex single unit responses are recorded using a planar, Utah style array in layer 3/4. An important and understudied aspect of INS is the effect of laser energy and interstimulus interval changes on evoked firing rate responses; a so-called dose-response curve. We begin by specifying predicted and predictor values. Dose-response relationships were measured by predicting maximum firing rates in response to applied INS energy (E) and inter-pulse intervals (ISI). As we suspect an interaction between E and ISI, an interaction term of E*ISI was incorporated. Therefore, the model was defined as:

$$\text{max}(FR)=\alpha +\beta_{1}E+\beta_{2}ISI+\beta_{3}(E*ISI)+\epsilon$$

An important aspect of this study was that rats underwent chronic recordings through the duration of the lifetime of the implant. It almost a certainty that stimulation and recording quality will change over the lifetime of the devices due to neural adaptation to stimulation(Falowski et al., 2011) and glial response and encapsulation of the devices(Van Kuyck et al., 2007; Woolley et al., 2013). This experimental paradigm is thus complicated by potentially meaningful repeated measures within subject variability. Furthermore, slight differences in electrode and optrode placement between rodents could create a heterogeneity in the receptive fields of recorded neurons(Vasquez-Lopez et al., 2017), representing a potentially meaningful between-subject variance.

### Hierarchical Structures Capture Latent Variables

Models in both Bayesian and frequentist paradigms capture these within and between subject variances by adding hierarchical structure to the model. From the Bayesian perspective, hierarchical models are defined by allocating hyperparameters on the prior which encode within and between group variances in the model, with each hyperparameter containing hyperprior distributions. Graphically, this is organized in Fig 5B. Bayesian and frequentist hierarchical models share similar roots, with particular hyperprior distributions in Bayesian paradigms becoming proportional to frequentist random effects models.

While this appears to be a herculean task in data modeling, PyMC allows for declarations of hierarchical models, as shown in Code Snippet 4:

Code Example 4: Creating a hierarchical regression model


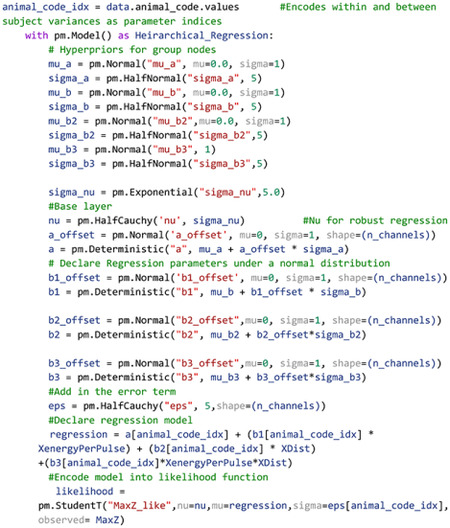


Owing to the scarcity of thalamocortical INS data, we assigned noninformative, wide spread normal distributions on the priors and hyperpriors so as to let the data speak for itself. We also utilized a student-T distribution as the likelihood function to accommodate outliers in a modification known as “robust regression”(Kruschke, 2014). Student-T distributions have tails which are not bounded by the exponential function, meaning that extreme values have less impact or skew on the posterior distribution. Half-Cauchy distributions are placed on the error term and Student-T normality parameter *v*. Half-Cauchy distributions are advantageous in learning scale parameters from the data in hierarchical models (Gelman, 2006; Polson and Scott, 2012).

It is important to validate that our model and data generating functions indeed represent the observed data. Sensitivity analyses and posterior predictive checks thus can be performed to ensure the model chosen is the one that best describes the observed data. Sensitivity analyses were performed by varying prior variance and comparing models which were nominal or natural log transformed with normal and student-T likelihood functions. Model comparisons can be performed in many ways, but a common paradigm is the leave-one-out cross validation (LOO)(Gelman et al., 2014). LOO consists of partitioning data into training and test sets and iteratively fitting the model under test with training data and testing out of sample fits with test data. Models are then ranked using the expected log pointwise predictive density (ELPD) measure:

$$\text{ELPD}=\sum_{i=1}^{k}\int dy_{i}p_{t}{\bar{y}}_{l}\text{log}\left(p\left({\bar{y}}_{l}|y\right)\right)$$

where *p*_*t*_, *y*_*i*_ are unknown distributions representing the true data generating function for estimates of true posterior predictive function ($\bar{y}|y$) from observed data *y*(Vehtari et al., 2017). In general, larger values of ELPD represent better out of sample fits indicative of a better model conditioned on observed data. We can then use standard errors between the model with the best ELPD (dse) and all competing models to rank all models to observed data. Importantly, these metrics should be understood only in the context of a model relative to other models, and not a global predictor of model validity. Observations of posterior fits to the data using posterior predictive fits and Bayesian p-values should be utilized on the final model to determine model fit. This seemingly complex model comparison can be quickly and easily done in PyMC with the following commands:

Code Example 5: Model Comparisons


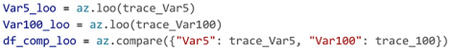


Model comparison results are given in Table 1. Similar to the simple regression above, the log transformed model provided much better fits to observed data than non-log transformed models. Interestingly and instructively, moderately informative priors (variance 5) outperformed noninformative priors (variance 100), suggesting that constraining prior variance can have predictive power in inference. Posterior predictive checks on the winning model show good fits to observed data with a Bayesian p-value near 0.5.

We can now perform inference on our multiregression model. It was found (Fig 5C) that *α* was significantly above 0 (MAP = 2.2, 95% HDI does not cross 0) suggesting that basal firing rates of recorded neurons were typically above 0 as expected. It was also seen that maximal firing rates were significantly dependent on applied INS energy (*β*_1_MAP = 0.58, HDI does not cross 0) with increases in INS energy leading to larger evoked maximal firing rates. The relative spread of the 95% HDI on *β*_1_ of 0.27–0.88 suggests a heterogeneity in neuron dose-response characteristics that can be explored more. Somewhat surprisingly, there was no significant effect of ISI on maximum firing rates (*β*_2_ MAP = −0.055). The relative spread across 0 of −0.45 to 0.3 suggests that extreme values of ISI might potentially have an effect, with smaller ISIs causing neural integration of singular INS pulses into a singular, large pulse. However, that cannot be determined given the INS parameters used in this study. Also surprisingly, there was no significant effect of Energy-ISI interactions (*β*_3_ MAP = 0.028), suggesting that INS energy is the primary mediator of evoked firing rates.

### Bayesian ANOVAs

Comparison of differences between groups is another routine statistical procedure used when predictor variables are nominal or categorical in nature or a mixture of metric and categorical predictors. The frequentist treatment of these experimental designs largely uses analysis of variances methods, namely ANOVA for categorical predictors and, more generally, ANCOVAs for categorical predictors with metric covariates. ANOVAs are models that take the form of:

$$y=\alpha +\sum_{i}\beta_{i}x_{i}$$

where *β*_*i*_, *x*_*i*_ are the parameters corresponding to nominal predictor class *i*, *α* is the offset or bias parameter, and *y* is the metric dependent variable. ANOVA parameters and class values *β*_*i*_, *x*_*i*_ are treated differently than the regression case, as *x*_*i*_ are categorical as opposed to continuous, metric values. As such *x* categories are recast into “one-hot” encoded vectors $\vec{x}=\left[x_{0},x_{1},\ldots ,x_{i}\right]$ in which only a singular value in an array can have a value of 1 and all other elements are cast to 0, allowing for binary indication of a given class among a group of classes. If an individual value falls into group *j*, for example, ${\vec{x}}_{i\neq j}=0$, ${\vec{x}}_{i=j}=1$. The coefficients *β*_*i*_ then encodes the change in dependent variable *y* from inclusion of datapoint *x* in category *i*. Importantly, deflections from baseline are constrained such that ∑_*i*_ *β*_*i*_ = 0. Both Bayesian and frequentist ANOVA models treat *β*_*i*_ parameters as group deflections about the baseline level of the dependent variable.

ANCOVA is a modification to the ANOVA model to include a metric covariance term:

$$y=\alpha +\sum_{i}\beta_{i}x_{i}+\beta_{co},x_{co}$$

where *β*_*co*_, *x*_*co*_ are the parameters corresponding to metric predictors. Metric predictors terms are valuable in accounting for within group variance which is attributable to some other metric measurable variable, such as decreased firing rates in response to an applied stimulus found in a class of aged animals.

Bayesian analogues of ANOVA and ANCOVA can be easily defined in PyMC and are termed BANOVA and BANCOVA (Fig 5A) respectively to distinguish models from their frequentist counterparts. Traditional ANOVAs make two key assumptions; that underlying data is normally distributed and a homogeneity of variance among groups. To account for these assumptions, normal distributions are placed on prior parameter and observed data distributions and a uniform distribution prior is placed on observed data variance *σ*_*y*_. Importantly, observed data distributions should be assessed to assure distributions are normally distributed. While not strictly an ANOVA-like structure, an advantage of Bayesian approaches is the ability to create models which handle arbitrary distributions. While traditional ANOVAs also assume independent group variances, the relative shared influence between groups can be learned from the data by imposing a hyperprior on group variance *σ*_*β*_(Gelman, 2006). As with any prior distributions, selection of *σ*_*β*_ should be informed by prior inspection of the data. A Half-Cauchy distribution is once again chosen as it weakly informative and allows for extreme values if data dictates(Gelman, 2006; Polson and Scott, 2012). Setting *σ*_*β*_ to a large constant replicates a traditional ANOVA.

**Figure 6:**
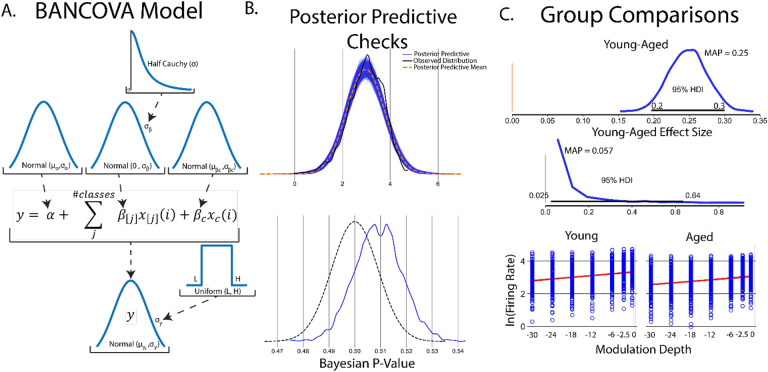
An example of Bayesian inference using ANOVA-like models. A. General schematic of BANOVA/BANCOVA models. Traditional ANOVAs have two key assumptions; normality of group data and homogeneity of variance. Normality of group data is imposed in BANOVA-like models as normal distributions around group parameters with homogeneity of variance encoded as a uniform distribution around posterior variance term *σ*_*y*_. Traditional ANOVAs assume a fixed variance on group parameter values *σ*_*β*_, imposing the constraint that each group is estimated independently from each other group. A uniquely Bayesian approach is to instead learn *σ*_*β*_ values from the data itself by placing a distribution on *σ*_*β*_. B. Posterior predictive checks suggest posterior distributions show good fit in mean and variance to observed data. C. Once posterior distributions are calculated, group comparisons can be easily done by subtracting young and aged posteriors to yield a contrast distribution. It is found that firing rates across all modulation depths are significantly higher in aged vs young rodents (contrast MAP = 0.25, 95% HDI does not overlap 0). Another unique feature of Bayesian approaches is the ability to assess distributions on effect size. In this BANCOVA, while group differences are significant, their relative effective size is small but significant (effect size MAP = 0.057, 95% HDI does not cross 0) suggesting marginal impact of age on firing rates elicited from SAM stimuli. Finally, metric covariates of firing rate in response to varying SAM depth in young and aged groups can be plotted as regressions superimposed on raw data.

As a guiding example, consider a similar experiment to that done in simple linear regression. In this experiment, we aim to understand age-related changes in IC auditory processing of sinusoidal amplitude modulated sounds. This experiment consisted of two groups of young (animals < 6 months in age) and aged (animals > 22 months in age). SAM stimuli at increasing modulation depths were played to the animals with evoked single unit responses recorded from IC. As seen in the regression experiment (Fig 2), there is a significant increase in evoked firing rate with increased modulation depth in young animals. As such, it should be included in comparison between the two groups. Taken together, this suggests BANCOVA will serve as an appropriate model. BANCOVAs are inherently hierarchical(Gelman, 2005; Kruschke, 2014) (Fig 6A) to allow for between subject variances to be represented in the prior if these variances mutually inform one another. Setting this hyperprior to a constant creates a model analogous to a frequentist ANCOVA(Kruschke, 2014). The formation of the BANCOVA is again relatively straightforward:

Code Example 6: Creating a Bayesian ANCOVA


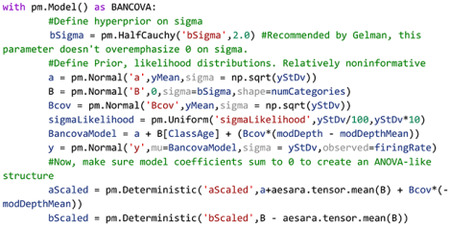
 with inference made in the exact same way as the previous models.

After model sampling, posterior sampling checks were performed to ensure posterior distributions adhere well to observed data. Posterior predictive distributions show good qualitative fit to observed firing rate data with Bayesian p-values centered around 0.51, suggesting good model fits to observed data (Fig 6B). Comparisons between groups is simple once posterior distributions are obtained. All that needs to be done is to measure differences between aged and young group parameter posteriors (Fig 6C), encoding relative influence of young and age groups on firing rates. Aged and young contrasts show significantly elevated firing rates in young rats across all SAM stimuli (Young-aged difference MAP = 0.25, 95% HDI excludes 0). Another advantage of Bayesian inference is the ability to observe the distribution, and thus the most likely value and spread of effect size. In this analysis, the effect of age in SAM stimulus processing is significant but small (effect size MAP =0.058, 95% HDI excludes 0) but with a wide spread of effect (95% HDI between 0.025–0.64) suggesting variable temporal acuity between rodent subjects. Finally, firing rates vs SAM amplitude depth for each class are plotted with *y* = *α* +*β*_*young*/*age*_*x*_*young/age*_ + *β*_*cov*_*x*_*cov*_ superimposed.

### Multiple Comparisons in Bayesian Inference

In traditional frequentist analyses, corrections for multiple comparisons are necessary in order to ensure that maximum Type I errors (false positives) are constrained to a maximum of 5% (*α* = 0.05). With Bayesian inference, a posterior distribution across all parameters is obtained which remains unchanged no matter how many comparisons are made(Kruschke, 2014). Furthermore, frequentist type I errors are classically defined in the context of rejection of a null hypothesis. Bayesian inference is not strictly concerned with rejection of a null hypothesis, instead weighing competing hypotheses given observed data. Bayesian models are not immune to making false conclusions about data. These errors, called type M for errors in magnitude and type S for errors in sign occur when outliers in data exert too much influence on inference. These errors can be controlled by proper choice of priors or by building hierarchical models (Fig 5A, Fig 6A) which can account for outliers by pulling parameters towards group means when evidence is small and allowing parameters with good evidence to remain in a phenomenon called partial pooling implicit to hierarchical structures(Gelman et al., 2009).

## Discussion

Bayesian inference approaches present a powerful statistical tool which encourages deep and meaningful exploration of data and allows for presentation of data in intuitive and transparent ways. In this tutorial, we demonstrate the ease by which Bayesian inference can be performed across a wide variety of experimental designs and provide source code which can be modified to accommodate neuroscientific experiments using all free and open source tools. We intentionally used the base PyMC toolchain in order to explicitly show Bayesian model creation. However, there are PyMC plugin tools such as Bambi (Capretto et al., 2022) which can facilitate creation of Bayesian models in single lines of code. An example of Bambi-enabled model creation is provided in our Bayesian inference toolbox.

### Tempering Expectations of Bayesian Inference

Despite the enthusiasm of some Bayesian advocates, Bayesian inference is not a panacea. It is subject to similar problems as frequentist NHST, in that models can be used which do not adequately fit underlying data statistics or priors can be chosen which dominate model performance and deemphasize observed data. However, Bayesian approaches support and encourage model transparency, requiring researchers to declare model priors and posteriors while encouraging continued discussion of inference on data as opposed to stopping if a p-value is below an arbitrary threshold. A second caveat is that running MCMCs can be slower than frequentist approaches, with run times sometimes in minutes as opposed to seconds. However, time increases are not astronomical and can be further reduced to levels similar to frequentist approaches by using GPU computing or using programs such as JASP(Love et al., 2019) which utilize a C backend to speed up computation.

### The Controversy of the Prior

The prior is arguably the most contentious aspect of Bayesian inference, with arguments that the prior unduly influences decisions on data. It is absolutely possible to have priors that distort posterior distributions into poor inference. Similar arguments can be levied at Frequentist approaches which perform similar distortions on decision metrics, such as applying ANOVA tests when underlying data is not normal. Often times, these mistakes are not done out of malevolence, but due to the modern framework of how statistics is performed. We argue that having to consider what prior to use, and thus what one’s assumptions are, what distributions are physiologically relevant, and the distributions of observed data will help to prevent errors in statistical modeling while creating greater transparency in how conclusions on data are drawn.

### Decisions with Bayes Factors

Some studies which utilize Bayesian inference use a decision metric called a Bayes’ factor, which is a measurement of the ratio of marginal likelihoods of two competing models providing log likelihood of evidence for one model over another(Johnson et al., 2023). We intentionally chose not to utilize Bayes’ factor metrics because, in the authors’ opinions, they reduce inference to evaluation of a single metric over an arbitrary threshold, as opposed to analysis over posterior distributions of observed data. Furthermore, certain prior declarations yield undefined Bayes’ factors(Gelman and Rubin, 1995) potentially encouraging using suboptimum models in order to provide arbitrary decision metrics.

### Bayesian and Frequentist Approaches: A Wholistic Approach to Inference

Following in the steps of Bayarri and Berger(Bayarri and Berger, 2004), data analysis should not consist solely of Bayesian or frequentist approaches devoid of the other. There are certainly cases where frequentist approaches should be used, such as clinical trials where preregistration and proper protocol design can provide bounds on false-positive and false negative rates necessary for translation of medical therapeutics. Hybrid frequentist and Bayesian approaches can also provide richer insight into analyses where posterior distributions are unidentifiable or difficult to sample(Raue et al., 2013) or in identifying when improper models have been chosen(Berger et al., 1997). Bayesian ideas of posterior predictive checks and model comparisons can also be applied to frequentist NHST, many of which would help address problems of replication and data transparency. As frequentist approaches are often baked into the pedagogy of neuroscience and neural engineering, we aim for this tutorial to be a thorough introduction into the application of Bayesian statistics to help develop a toolkit which can be used for robust data analysis or in conjunction with previously established frequentist approaches. These models are also easily extendable into Bayesian analogs of logistic or multinomial regressions, gaussian mixture models, Bayesian time series analyses, among many more.

## Supplementary Material

1

## Figures and Tables

**Figure 4:**
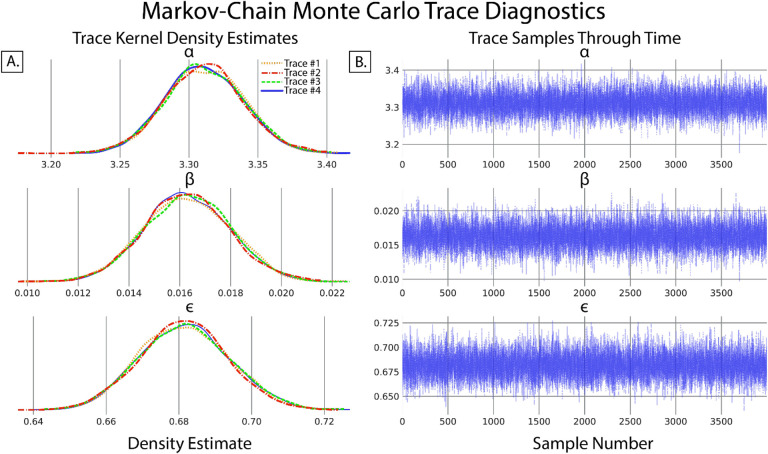
Evaluation of Markov-chain Monte Carlo (MCMC) chains can help diagnose ill fitting distributions. A. Kernel density estimates of the marginal posteriors corresponding to each of the regression parameters of each MCMC trace. Qualitatively, chain distributions should appear similar to each other, suggesting good convergence to target distributions. B. Time series plot of trace value vs sample number of marginal posteriors corresponding to each regression parameter. Qualitatively good traces should have a “fuzzy caterpillar” like shape, evident in all parameters of this model, indicative of good integration over the joint posterior distribution and effective sampling of the posterior.

**Table 1:** LOO Model comparisons and sensitivity analyses

Model	R	ELPD	DSE
*St Log Var 5*	1	−5337.48	0.00
*ST Log Var 100*	2	−5337.62	0.420867
*ST Log Var 0.5*	3	−5337.76	0.409773
*St Log Var 25*	4	−5338.15	0.492297
*ST Log Var 10*	5	−5338.18	0.300197
*ST Log Var 1*	6	−5338.26	0.331152
*N Log Var 10*	7	−5340.60	3.308668
*N Log var 1*	8	−5341.09	3.293779
*N log var 5*	9	−5341.16	3.296273
*N log var 0.5*	10	−5342.46	3.300550
*ST Semilog Var 1*	11	−5466.76	15.845916
*St Semilog var 5*	12	−5467.12	15.856552
*ST semilog var 10*	13	−5467.15	15.895646
*ST semilog var 0.5*	14	−5467.18	15.866405
*ST Var 1*	15	−15336.31	79.406629
*ST var 0.5*	16	−15355.67	80.415787
*St var 5*	17	−15355.67	80.415787
*N var 10*	18	−16119.11	82.384329
*N var 1*	19	−16132.23	83.549811
*N var 0.5*	20	−16154.55	84.262219

## Data Availability

The code/software described in the paper is freely available online at [URL redacted for double-blind review]. The code is available as Extended Data.

## Significance Statement

Bayesian inference has received renewed interest as an alternative to null-significance hypothesis testing for its interpretability, ability to encapsulate prior knowledge into current inference, and robust model comparison paradigms. Despite this renewed interest, discussions of Bayesian inference are often obfuscated by undue mathematical complexity and misunderstandings underlying the Bayesian inference process. In this article, we aim to empower neuroscientists to adopt Bayesian statistical inference by providing a practical methodological walkthrough using single and multi-unit recordings from the rodent auditory circuit accompanied by a well-documented and user-friendly toolkit containing regression and ANOVA statistical models commonly encountered in neuroscience.
