# Supplementary material for: Practical Bayesian Inference in Neuroscience: Or How I Learned To Stop Worrying and Embrace the Distribution
Source: bioRxiv. 2023 Nov 21:2023.11.19.567743. Preprint. [Version 1] doi: 10.1101/2023.11.19.567743 (PMC10690186; doi:10.1101/2023.11.19.567743)
Supplement: Supplementary file 1 [file NIHPP2023.11.19.567743V1-supplement-1.pdf]

bioRxiv preprint doi: https://doi.org/10.1101/2023.11.19.567743; this version posted November 21, 2023. The copyright holder for this preprint (which was not certified by peer review) is the author/funder, who has granted bioRxiv a license to display the preprint in perpetuity. It is made available under aCC-BY 4.0 International license.

Supplementary Material Outline

1. **Materials and Methods**

   1.1. Introduction
   1.2. Disruption of Temporal Processing in the Inferior Colliculus Due to Aging
   1.3. Thalamocortical Infrared Neural Stimulation

2. **An Example of Bayesian Analysis Reporting Guidelines**

3. **Supplementary Figures**

4. **Supplementary References**

bioRxiv preprint doi: https://doi.org/10.1101/2023.11.19.567743; this version posted November 21, 2023. The copyright holder for this preprint (which was not certified by peer review) is the author/funder, who has granted bioRxiv a license to display the preprint in perpetuity. It is made available under aCC-BY 4.0 International license.

1. Materials and Methods

Bayesian inference was performed on a range of data typical to neuroscience experiments. Regression models, ANOVA models, and group comparisons are performed on single-unit activity recorded from inferior colliculus (IC) neurons in response to auditory stimuli in young and aged rats(Palombi et al., 2001; Simon et al., 2004; C.F et al., 2012; Herrmann et al., 2017). Random-effects regression models are performed on single units recorded in the auditory cortex (A1) using high-density recording arrays in response to infrared neural stimulation(Izzo et al., 2007; Cayce et al., 2011, 2014; Coventry et al., 2023) of the medial geniculate body (MGB). To underscore that meaningful Bayesian inference does not require cluster computing or extensive computational resources, all computations were performed on an MSI GS-66 laptop with an Intel i7 processor with an Nvidia RTX2070 GPU. Our inference programs are CPU-bound, not requiring any GPU resources. Computations can be performed on most modern CPUs, but accelerate with more CPU threads and cores and parallelization on GPUs. All surgical procedures used in this study were approved by [redacted for double-blind review].

1.1 Disruption of Temporal Processing in the Inferior Colliculus Due to Aging

The inferior colliculus (IC) is the major integrative center of the auditory pathway, receiving excitatory inputs from ventral and dorsal cochlear nuclei, excitatory and inhibitory inputs from the lateral and medial superior olivary complex(Kelly and Caspary, 2005) and inhibitory inputs from superior paraolivary nucleus and the dorsal and ventral nuclei of the lateral lemniscus(Cant and Benson, 2006; Loftus et al., 2010). The IC encodes auditory information through hierarchical processing of input synaptics with local IC circuitry(Caspary et al., 2002; Rabang et al., 2012; Grimsley et al., 2013; Coventry et al., 2017). Age-related changes in auditory processing primarily arise as deficits in temporal processing(Frisina and Frisina, 1997; Parthasarathy et al., 2010; Parthasarathy and Bartlett, 2012; Herrmann et al., 2017). This dataset is composed of single unit responses recorded from young (Age≤ 6 months) and aged (age ≥ 22 months) Fisher 344 rats. Auditory brainstem responses were recorded from animal subjects a few days prior to surgery to ensure hearing thresholds were typical of the rodent's age. Single unit recordings were performed in a 9'x9' double-walled, electrically isolated anechoic chamber (Industrial Acoustics Corporation). Animals were initially anesthetized via a bolus injection of ketamine (VetaKet, 60-80 mg/kg) and medetomidine (0.1-0.2 mg/kg) mixture via intramuscular injection. Oxygen was maintained via a manifold and pulse rate and blood oxygenation monitored through pulse oximetry. Supplemental doses of ketamine/medetomidine (20 mg/kg ketamine, 0.05 mg/kg medetomidine) were administered intramuscularly as required to maintain surgical plane of anesthesia. An incision was made down midline and the skull exposed. Periosteum was resected and a stainless steel headpost was secured anterior to bregma via 3 stainless steel bone screws. A craniectomy was made above inferior colliculus (-8.5 anterior/posterior, 1 mm medial/lateral from bregma). A single tungsten electrode was advanced dorsally towards the central nucleus of the inferior colliculus (ICC) during which bandpass noise (200 ms, center frequencies 1-36kHz in five steps per octave, 0.5 octave bandwidth) was delivered. ICC was identified based on short-latency driven responses to bandpass noise search stimuli with ascending tonotopy and narrowly tuned responses to pure tones of varying frequencies. Once neurons were identified, responses from 5-10 repetitions of sinusoidal amplitude-modulated tones (750 ms tone length, modulation depth between -30 to 0 dB) were recorded using a preamplifying headstage (RA4PA, Tucker-Davis Technologies) and discretized at a sampling rate of 24.41 kHz (RZ-5, TDT). Sinusoidal amplitude-modulated tones were defined as:

$$s(t) = A[1 + m * cos(2\pi f\_m\, t + \varphi)] * n(t)$$

bioRxiv preprint doi: https://doi.org/10.1101/2023.11.19.567743; this version posted November 21, 2023. The copyright holder for this preprint (which was not certified by peer review) is the author/funder, who has granted bioRxiv a license to display the preprint in perpetuity. It is made available under aCC-BY 4.0 International license.

where m is modulation depth ranging between 0.032-1 (-30 – 0 dB), f_m the modulation frequency, φ the reference phase of the modulator, A the scaling factor for stimulus sound level, and $n(t)$ the broadband noise stimulus. Single units were filtered between 0.3 and 5 kHz. Offline spike sorting was performed using OpenExplorer (TDT).

1.2 Thalamocortical Infrared Neural Stimulation

Infrared neural stimulation (INS) is an optical technique using coherent infrared light to stimulate nerves and neurons without the need for genetic modification of the target or direct contact with tissue that offers spatially constrained activation above electrical stimulation(Wells et al., 2005; Izzo et al., 2007; Cayce et al., 2011, 2014; Coventry et al., 2020, 2023). In this study, rats were chronically implanted in A1 with 16 channel planar Utah-style arrays (TDT, Alacua FL) and stimulating optrodes in the medial geniculate body of auditory thalamus (Thor Labs, Newton NJ). Rodents were initially anesthetized with a bolus injection of a ketamine (80 mg/kg) and medetomidine (0.2 mg/kg) cocktail. Oxygen was maintained via a manifold and pulse rate and blood oxygenation monitored through pulse oximetry. Supplemental doses of ketamine/medetomidine (20 mg/kg ketamine, 0.05 mg/kg medetomidine) were administered intramuscularly as required to maintain surgical plane of anesthesia. An incision was made down midline and the skull exposed. The periosteum was removed via blunt dissection and 3 stainless steel bone screws were placed in skull for headcap stability. An additional titanium bones crew was placed in skull to serve as a chronic ground and reference point for recording electrodes. Craniectomies were made above medial geniculate body (-6 anterior/posterior, -3.5 medial/lateral from bregma) and auditory cortex (-6 anterior/posterior, -5 medial/lateral from bregma). Fiber optic stimulating optrodes were placed in the midpoint of MGB (-6 dorsal/ventral from dura) and affixed to the skull using UV-curable dental acrylic (MidWest Dental). A 16 recording channel planar array was putatively placed in layers 3/4 of auditory cortex, with placement confirmed by short-latency high amplitude multiunit activity elicited from band pass noise (200 ms, center frequencies 1-36kHz in five steps per octave, 0.5 octave bandwidth) test stimuli. Recording electrodes were sealed onto the headcap. Animals were allowed to recover for 72 hours prior to the beginning of the recording regime. All recordings were performed in a 9'x9' electrically isolated anechoic chamber. During recording periods, animals received a intramuscular injection of medetomidine(0.2 mg/kg) for sedation. Optical stimuli were delivered from a 1907 nm diode laser (INSight open source optical stimulation system) coupled to the optrode with a 200 μm, 0.22 NA fiber (Thor Labs FG200LCC). Laser stimuli were controlled via a RX-7 stimulator (TDT) and consisted of train stimuli with pulse widths between 0.2-10 ms, interstimulus intervals between 0.2-100 ms and energy per pulse between 0-4 mJ. Applied laser energies were randomized to limit effects from neural adaptation with 30-60 repetitions per pulse width/interstimulus interval combinations. Signals from recording electrodes were amplified via a Medusa 32 channel preamplifier and discretized and sampled at 24.414 kHz with a RZ-2 biosignal processor and visualized using Open-Ex software (TDT). Action potentials were extracted from raw waveforms via real-time digital band-pass filtering with cutoff frequencies of 300-5000 Hz. Single units were extracted offline via superparamagnetic clustering in WaveClus (Quiroga et al., 2004). Studies were performed to assess the dose-response profiles of optically-based deep brain stimulation over the span of several months. As each electrode recorded diverse populations of neurons which are potentially subject to change due to electrode healing in, age of the device, and adaptation to the stimulus, a within subjects, repeated measures regression model was warranted. Bayesian hierarchical regressions can easily deal with complex models such as these.

2. An Example of Bayesian Analysis Reporting Guidelines

bioRxiv preprint doi: https://doi.org/10.1101/2023.11.19.567743; this version posted November 21, 2023. The copyright holder for this preprint (which was not certified by peer review) is the author/funder, who has granted bioRxiv a license to display the preprint in perpetuity. It is made available under aCC-BY 4.0 International license.

Bayesian Analysis Reporting Guidelines (BARG)(Kruschke, 2021) was initially proposed to promote transparent and reproducible Bayesian statistics reporting. While initially devised for social and psychological sciences, we adapted the BARG to suit neuroscientific data.

1. **Bayesian Model Descriptions and Sensitivity Analyses.** This report follows the guidelines for reporting of Bayesian Analysis (BARG) (Kruschke, 2021) consisting of:

- Necessary software and source code directory
- Goals of the analysis
- Model descriptions and decision criterion
- Prior and hyperprior descriptions
- Sensitivity analyses for varying prior distributions
- Posterior and MCMC diagnostics

1.1 Necessary software and source code directory

BARG: Step 2A, 6

This section describes the computational tools used for statistical analyses, including CPU and GPU use. For example:

Bayesian modeling was performed using Python 3.6.8 on a Razer Blade 15 Laptop with an Intel Core i7 processor (6 cores) and an Nvidia RTX2070 GPU. Models were implemented in PyMC version 4.11.5 (Salvatier et al., 2016), a probabilistic programming module in the Python environment. All source code is available at this paper's github repository (Link to software). All source data is available at this article's open science framework repository (Link to Data).

1.2 Goals of the Analyses

This section serves to establish goals of the analyses, brief description of the statistical models used and validation of Bayesian approaches.

BARG: Preamble

The goal of Bayesian regression analyses is to infer a linear relationship within inferior colliculus single unit firing rates resulting from changes in depth of sinusoidal amplitude modulated stimuli. While this is normally established using frequentist linear regression methods, Bayesian approaches allow for flexible and explicit model descriptions which provide rich and descriptive inference and quantification of uncertainty in measurement of single unit activity. Inference is completed using direct probability measures on posterior distributions as opposed to less intuitive and difficult to interpret p-values. Bayesian approaches are also data driven and account for previous knowledge to be encoded as prior distributions (see section 1.3).
The regression model utilized is:

$$\ln(FR) = \alpha + \beta * m + \epsilon$$

where FR is the mean evoked firing rate. Firing rate functions were calculated from recorded peristimulus time histograms. Parameter β quantifies the effect of modulation depth (m) on evoked firing rates respectively. The α parameter describes the model intercept and quantifies subthreshold spontaneous activity and the ϵ quantifies model error.

1.3 Prior Selection

Priors and rational for prior choice is described in this section

There is significant data detailing inferior colliculus responses to SAM stimuli from our lab and the auditory neuroscience community writ large(Citations redacted for double blind review). However,

bioRxiv preprint doi: https://doi.org/10.1101/2023.11.19.567743; this version posted November 21, 2023. The copyright holder for this preprint (which was not certified by peer review) is the author/funder, who has granted bioRxiv a license to display the preprint in perpetuity. It is made available under aCC-BY 4.0 International license.

the role of modulation depth on IC firing rates is understudied. As observations of single unit IC activity tends towards normal distributions, normal likelihood and prior distributions were chosen. Normal distributions also have the advantage of being moderately informative, refraining from undue influence on the posterior from the prior, allowing data to “speak for itself.”

1.4 Posterior Decision Rules

This section details the decision rules used in inference (ROPE+HDI, Bayes factors, etc).

Inference was performed on posterior distributions with credible regions (analogous to frequentist confidence intervals) defined as a highest density interval (HDI) of 95% of parameter maximal *a posteriori* density (MAP) parameter estimates which represent the most probable value of the coefficient. MAP estimates are analogous to maximum likelihood estimation found in frequentist approaches. This allows for the quantification of parameter uncertainty as variance observed in posterior parameter distributions, with narrow HDIs representing more certain estimates. It is customary to define a region of practical equivalence (ROPE) if prior information dictates that incremental parameter changes are effectively the same. As we lack prior knowledge to inform the choice of a prior rope, we take an agnostic approach that any change seen is worth investigating and thus ROPEs are not presented. An effect was deemed significant if it’s 95% HDI did not overlap with 0, in line with proposed decision rules typical of Bayesian inference(Kruschke, 2011, 2014).

1.5 Final Model

This section details the final model after prior and posterior sensitivity analyses. Helpful to include a descriptive figure of the inference model

Posterior predictive checks and sensitivity analysis were performed to titrate the best performing models as measured against observed data (Section 3). The final regression model is schematized in figure S1. Final models included deterministic nodes at outputs of prior nodes to prevent NUTS from becoming stuck in regions of the sampling space which are difficult to explore [1].

1.6 Model Sensitivity analyses

BARG: Step 3A,C

This section details the methods and results of any model sensitivity analyses. As an example, Model sensitivity analyses from hierarchical linear regression are given below.

To evaluate the dependance of hyperprior and prior parameters on Bayesian hierarchical linear regression, leave one out (LOO) cross validation(Gelman et al., 2014). A series of models were evaluated with model variances varied to test sensitivity of each model. Initial data analyses suggested that natural-log transformations of the dependent variable (firing rate) produced distributions which are better modeled as normal distributions. To this end, hierarchical models under test were as follows:

MODEL NAME	MODEL
REGRESSION	$FR = \alpha + \beta_1 * Energy + \beta_2 * ISI + \beta_3 * Energy * ISI + \epsilon$
SEMILOG REGRESSION	$ln(FR) = \alpha + \beta_1 * Energy + \beta_2 * ISI + \beta_3 * Energy * ISI + \epsilon$
NATURAL LOG REGRESSION	$ln(FR) = \alpha + \beta_1 * ln(Energy) + \beta_2 * ln(ISI) + \beta_3 * ln(Energy) * ln(ISI) + \epsilon$

Table S1: Regression models under test

bioRxiv preprint doi: https://doi.org/10.1101/2023.11.19.567743; this version posted November 21, 2023. The copyright holder for this preprint (which was not certified by peer review) is the author/funder, who has granted bioRxiv a license to display the preprint in perpetuity. It is made available under aCC-BY 4.0 International license.

For each model, the variance hyperprior was varied to assess the impact of prior parameters on posterior predictions. Prior classes were defined as: informative (variance ≤ 1), moderately informative (variance $= 5$), and weakly informative (variance ≥ 10). Primary metrics for model comparison were expected log pointwise predictive density (ELPD), defined as(Vehtari et al., 2017):

$$elpd = \sum_{i=1}^{k} \int dy_i p_t \bar{y}_i \log\left(p(\bar{y}_i|y)\right)$$

where p_t, y_i are unknown distributions representing the true data generating function for estimates of true posterior predictive function $(\bar{y}|y)$ from observed data y. Estimated p_t, y_i distributions are obtained via cross validation during LOO analysis. In general, higher values of ELPD are a result of higher out of sample predictive fit indicative of a better model. Weight values generated by LOO cross validation were also analyzed and predict the probability of each model given observed data. Finally, we observed the standard error of the ELPD estimate (SE), and the difference between the model with highest ELPD and every other model (dSE) with dSE of the top model set to 0.00 by definition. All LOO calculations were performed *post hoc* with the python package arviz, a plugin for PyMC.

MODEL	R	ELPD	WEIGHT	SE	DSE
**ST LOG VAR 5**	1	-5337.48	2.046623e-01	46.220458	0.00
**ST LOG VAR 100**	2	-5337.62	1.763745e-01	46.227682	0.420867
**ST LOG VAR 0.5**	3	-5337.76	1.552051e-01	49.173347	0.409773
**ST LOG VAR 25**	4	-5338.15	1.058393e-01	46.358847	0.492297
**ST LOG VAR 10**	5	-5338.18	9.996540e-02	46.141502	0.300197
**ST LOG VAR 1**	6	-5338.26	9.238175e-02	49.030330	0.331152
**N LOG VAR 10**	7	-5340.60	7.103823e-02	49.024680	3.308668
**N LOG VAR 1**	8	-5341.09	4.291607e-02	48.985814	3.293779
**N LOG VAR 5**	9	-5341.16	3.978488e-02	89.737613	3.296273
**N LOG VAR 0.5**	10	-5342.46	1.183257e-02	89.930943	3.300550
**ST SEMILOG VAR 1**	11	-5466.76	4.359604e-37	84.933022	15.845916
**ST SEMILOG VAR 5**	12	-5467.12	3.535240e-37	89.043113	15.856552
**ST SEMILOG VAR 10**	13	-5467.15	5.622764e-37	85.266895	15.895646

bioRxiv preprint doi: https://doi.org/10.1101/2023.11.19.567743; this version posted November 21, 2023. The copyright holder for this preprint (which was not certified by peer review) is the author/funder, who has granted bioRxiv a license to display the preprint in perpetuity. It is made available under aCC-BY 4.0 International license.

**ST SEMILOG VAR 0.5**	14	-5467.18	3.483572e-37	85.266895	15.866405
**ST VAR 1**	15	-15336.31	0.000000e+00	49.465018	79.406629
**ST VAR 0.5**	16	-15355.67	0.000000e+00	49.509352	80.415787
**ST VAR 5**	17	-15355.67	0.000000e+00	49.487001	80.415787
**N VAR 10**	18	-16119.11	0.000000e+00	49.510419	82.384329
**N VAR 1**	19	-16132.23	0.000000e+00	49.524316	83.549811
**N VAR 0.5**	20	-16154.55	0.000000e+00	49.514661	84.262219

Table S2: LOO model comparison results for the Bayesian hierarchical regression models. Var: Prior variance parameter, log: log predictor and predicted variable model. semilog: semilog predictor model. ST: Student T Likelihood models. N: Normal likelihood models

1.7 Posterior and MCMC Diagnostics

This section details model diagnostics surrounding the Bayesian Inference procedure.

BARG: Step 1E, 2A-D, 3A,C

1.7.1 Choice of MCMC method

This section details the choice of Markov-chain Monte Carlo model used. Many are available, but NUTS is the most common.

For sampling, the Hamiltonian-based MCMC method no U-turn sampling (NUTS)**(Hoffman and Gelman, 2011)** was used. NUTS presents a modification of general Hamiltonian Monte Carlo samplers and presents an efficient sampler for hierarchical and high-dimensional models at the cost of slower sampling times. Regression models ran 4 simultaneous chains with 2000 burn in samples and 4000 iterations with a 90% target inclusion probability.

MCMC Diagnostics

Energy transition plots were used to assess how well MCMC sampled the target posterior distribution of the best performing model as assessed by PSIS-LOO metrics which were compared between models**(Betancourt, 2017)**. NUTS sampling is based off dynamical systems modeling (Hamiltonian Monte Carlo) movement through the high entropy distributions towards a target distribution. MCMCs are modeled as dynamical systems with “position” and “momentum” associated with transition between states. This allows for a measurement of kinetic energy associated with the sampler. Efficiency in MCMC trajectory towards the target distribution can be assessed by comparing energy associated with the marginal energy distribution. The regression model displayed overlapping marginal energy and energy transition distributions (Fig S2) suggesting that sample to sample movement was nearly independent and indicative of efficient sampling of the target posterior distribution.

Furthermore, traces of sampled prior parameters in regression models suggest effective sampling of the posterior distribution (Fig S3). Furthermore, the Gelman-Rubin statistic, quantifying within

bioRxiv preprint doi: https://doi.org/10.1101/2023.11.19.567743; this version posted November 21, 2023. The copyright holder for this preprint (which was not certified by peer review) is the author/funder, who has granted bioRxiv a license to display the preprint in perpetuity. It is made available under aCC-BY 4.0 International license.

and between chain estimates and correlation was $\hat{\boldsymbol{r}} < \mathbf{1.05}$, indicative of convergence of marginal posterior parameter values**(Gelman and Rubin, 1992)**.

1.7.2 Posterior Predictive Checks

This section describes any posterior predictive checks performed and a description of the posterior predictive decision rule.

A key advantage of Bayesian-based inference approaches is the ability to directly and explicitly compare model fits to observed data. During model development, posterior predictive checks were performed by sampling from the posterior distribution (16,000 draws). Kernel density estimates of posterior predictive draws from the posterior distribution were compared to kernel densities of observed data. Goodness of fit was quantified using the Bayesian p-value**(Gelman et al., 2021)**. Similar to the frequentist p-value, the Bayesian p-value is also a measure of discrepancy, quantifying the probability that posterior predictive-based draws are more extreme than observed data. The Bayesian p-value is defined as:

$$p_B = \iint dy^r d\theta \; I_{T(y^r,\theta)\geq T(y^r,\theta)} p(y^r|\theta)p(\theta|y)$$

where I is the indicator function, $\boldsymbol{y}^r$ is the posterior predictive distribution and y is the posterior distribution. Similar to the posterior distribution, posterior predictive distribution and Bayesian p-values were estimated using NUTS. The closer the Bayesian p-value is to 0.5, the better the data sampled from the posterior distribute around the observed data.

Posterior predictive fits and Bayesian p-values for the hierarchical linear and multinominal regression models suggest excellent posterior predictive fits with $\overline{\boldsymbol{p}} = \mathbf{0.51}$ for the hierarchical linear regression model (Main article, Fig 4).

Prior and Posterior Trace plots

This section presents prior and posterior trace plots which are useful for diagnosing model fits.

BARG: Step 2B,C

Critical to the performance of HMC MCMC sampling is the convergence of sampling traces. Output trace plots display the chain of sampled values and the resulting kernel density estimates of sampled prior distributions. All sampled traces showed no divergences in sampling, suggesting that sampled traces were “well behaved”, providing accuate and effective sampling of the distribution. The Gelman-Rubin statistic, quantifying within and between chain estimates and correlation was $\hat{\boldsymbol{r}} < \mathbf{1.5}$ for all sampled traces thus showing good MCMC convergence. For clarity, traces are available on open science framework, with traces for the posterior presented in Figure S3. Traces were checked for characteristic sampling behavior**(Hoffman and Gelman, 2011)** with no pathological traces found in models.

bioRxiv preprint doi: https://doi.org/10.1101/2023.11.19.567743; this version posted November 21, 2023. The copyright holder for this preprint (which was not certified by peer review) is the author/funder, who has granted bioRxiv a license to display the preprint in perpetuity. It is made available under aCC-BY 4.0 International license.

3. Supplementary Figures

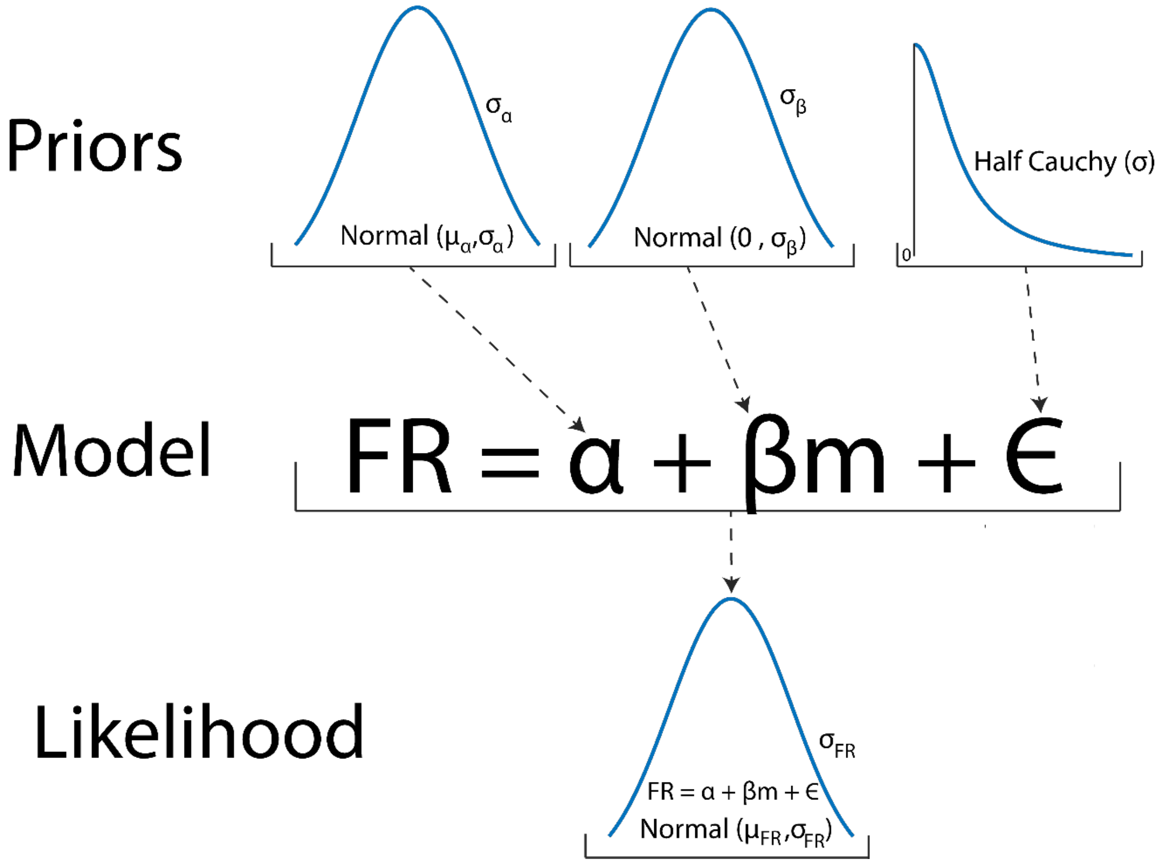

Figure S1: Schematic diagram of Bayesian Linear Regression

bioRxiv preprint doi: https://doi.org/10.1101/2023.11.19.567743; this version posted November 21, 2023. The copyright holder for this preprint (which was not certified by peer review) is the author/funder, who has granted bioRxiv a license to display the preprint in perpetuity. It is made available under aCC-BY 4.0 International license.

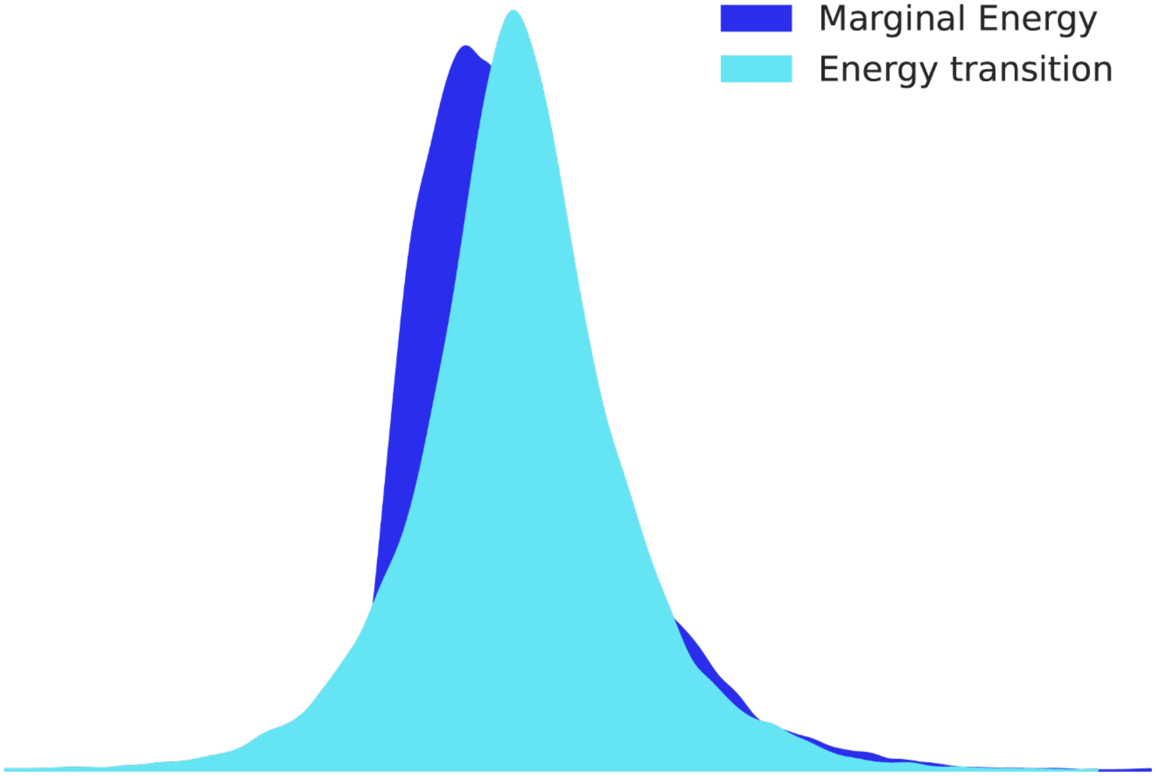

Figure S2: Energy transition diagram for Bayesian linear regression. Overlap of marginal energy with energy transition distributions suggests effective sampling of target distributions.

bioRxiv preprint doi: https://doi.org/10.1101/2023.11.19.567743; this version posted November 21, 2023. The copyright holder for this preprint (which was not certified by peer review) is the author/funder, who has granted bioRxiv a license to display the preprint in perpetuity. It is made available under aCC-BY 4.0 International license.

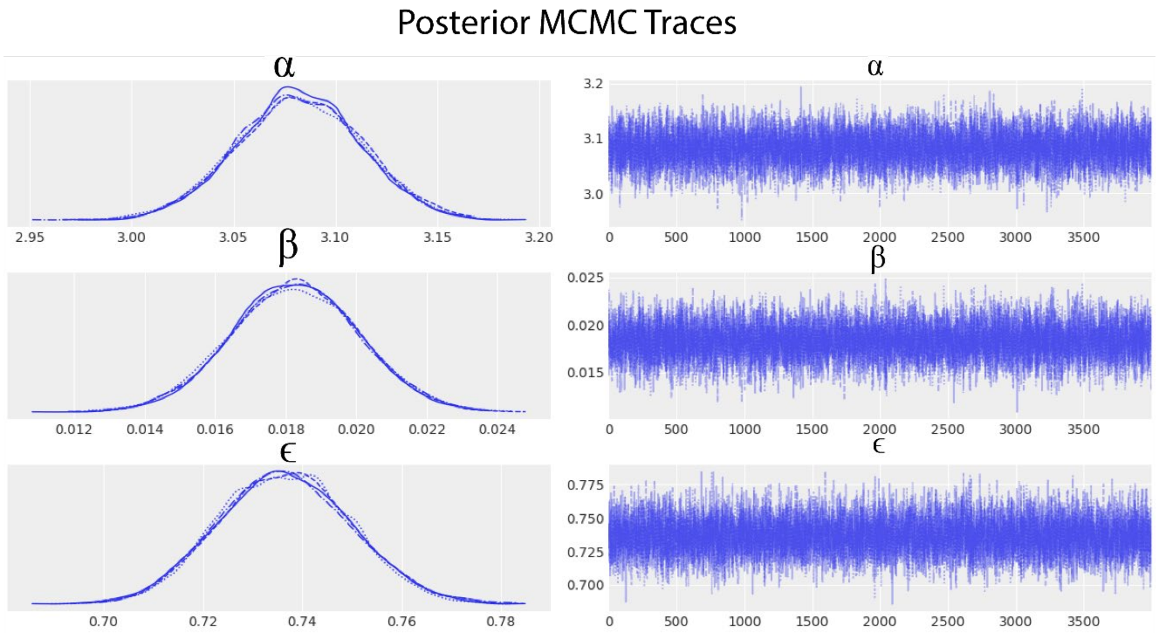

Figure S3: Bayesian regression posterior MCMC traces. MCMC chain distributions (left) built from HMC traces (right).

bioRxiv preprint doi: https://doi.org/10.1101/2023.11.19.567743; this version posted November 21, 2023. The copyright holder for this preprint (which was not certified by peer review) is the author/funder, who has granted bioRxiv a license to display the preprint in perpetuity. It is made available under aCC-BY 4.0 International license.

4. Supplementary References

Betancourt M (2017) A Conceptual Introduction to Hamiltonian Monte Carlo. Available at: https://arxiv.org/abs/1701.02434 [Accessed July 26, 2023].

Cant NB, Benson CG (2006) Organization of the inferior colliculus of the gerbil (Meriones unguiculatus): Differences in distribution of projections from the cochlear nuclei and the superior olivary complex. J Comp Neurol 495:511–528.

Caspary DM, Palombi PS, Hughes LF (2002) GABAergic inputs shape responses to amplitude modulated stimuli in the inferior colliculus. Hearing Research 168:163–173.

Cayce JM, Friedman RM, Chen G, Jansen ED, Mahadevan-Jansen A, Roe AW (2014) Infrared neural stimulation of primary visual cortex in non-human primates. NeuroImage 84:181–190.

Cayce JM, Friedman RM, Jansen ED, Mahavaden-Jansen A, Roe AW (2011) Pulsed infrared light alters neural activity in rat somatosensory cortex in vivo. NeuroImage 57:155–166.

C.F R, Parthasarathy A, Venkataraman Y, Fisher ZL, Gardner SM, Bartlett EL (2012) A computational model of inferior colliculus responses to amplitude modulated sounds in young and aged rats. Frontiers in Neural Circuits 6.

Coventry BS, Lawlor GL, Bagnati CB, Krogmeier C, Bartlett EL (2023) Spatially specific, closed-loop infrared thalamocortical deep brain stimulation. bioRxiv Available at: http://biorxiv.org/lookup/doi/10.1101/2023.10.04.560859 [Accessed October 12, 2023].

Coventry BS, Parthasarathy A, Sommer AL, Bartlett EL (2017) Hierarchical winner-take-all particle swarm optimization social network for neural model fitting. Journal of Computational Neuroscience 42:72–85.

Coventry BS, Sick JT, Talavage TM, Stantz KM, Bartlett EL (2020) Short-wave Infrared Neural Stimulation Drives Graded Sciatic Nerve Activation Across A Continuum of Wavelengths. In: 2020 42nd Annual International Conference of the IEEE Engineering in Medicine & Biology Society (EMBC). IEEE.

Frisina DR, Frisina RD (1997) Speech recognition in noise and presbycusis: relations to possible neural mechanisms. Hearing Research 106:95–104.

Gelman A, Carlin J, Stern H, Dunson D, Vehtari A, Rubin D (2021) Bayesian Data Analysis, 3rd ed. Boca Raton: Chapman and Hall/CRC.

Gelman A, Hwang J, Vehtari A (2014) Understanding predictive information criteria for Bayesian models. Stat Comput 24:997–1016.

Gelman A, Rubin DB (1992) Inference from Iterative Simulation Using Multiple Sequences. Statistical Science 7:457–472.

bioRxiv preprint doi: https://doi.org/10.1101/2023.11.19.567743; this version posted November 21, 2023. The copyright holder for this preprint (which was not certified by peer review) is the author/funder, who has granted bioRxiv a license to display the preprint in perpetuity. It is made available under aCC-BY 4.0 International license.

Grimsley CA, Sanchez JT, Sivaramakrishnan S (2013) Midbrain local circuits shape sound intensity codes. Front Neural Circuits 7 Available at: http://journal.frontiersin.org/article/10.3389/fncir.2013.00174/abstract [Accessed August 27, 2023].

Herrmann B, Parthasarathy A, Bartlett EL (2017) Ageing affects dual encoding of periodicity and envelope shape in rat inferior colliculus neurons Foxe J, ed. Eur J Neurosci 45:299–311.

Hoffman MD, Gelman A (2011) The No-U-Turn Sampler: Adaptively Setting Path Lengths in Hamiltonian Monte Carlo. Available at: https://arxiv.org/abs/1111.4246 [Accessed July 26, 2023].

Izzo AD, Suh E, Pathria J, Walsh JT, Whitlon DS, Richter C-P (2007) Selectivity of neural stimulation in the auditory system: a comparison of optic and electric stimuli. Journal of biomedical optics 12:021008.

Kelly JB, Caspary DM (2005) Pharmacology of the inferior colliculus. In: The inferior colliculus., 1st ed. New York, NY: Springer.

Kruschke JK (2011) Bayesian Assessment of Null Values Via Parameter Estimation and Model Comparison. Perspectives on Psychological Science 6:299–312.

Kruschke JK (2014) Doing Bayesian Data Analysis: A tutorial with R, JAGS, and stan, 2nd ed. Academic Press. Available at: http://www.indiana.edu/~kruschke/DoingBayesianDataAnalysis/.

Kruschke JK (2021) Bayesian Analysis Reporting Guidelines. Nat Hum Behav 5:1282–1291.

Loftus WC, Bishop DC, Oliver DL (2010) Differential Patterns of Inputs Create Functional Zones in Central Nucleus of Inferior Colliculus. J Neurosci 30:13396–13408.

Palombi PS, Backoff PM, Caspary DM (2001) Responses of young and aged rat inferior colliculus neurons to sinusoidally amplitude modulated stimuli. Hearing Research 153:174–180.

Parthasarathy A, Bartlett E (2012) Two-channel recording of auditory-evoked potentials to detect age-related deficits in temporal processing. Hearing Research 289:52–62.

Parthasarathy A, Cunningham PA, Bartlett EL (2010) Age-Related Differences in Auditory Processing as Assessed by Amplitude-Modulation Following Responses in Quiet and in Noise. Front Ag Neurosci 2 Available at: http://journal.frontiersin.org/article/10.3389/fnagi.2010.00152/abstract [Accessed March 13, 2023].

Quiroga RQ, Nadasdy Z, Ben-Shaul Y (2004) Unsupervised spike detection and sorting with wavelets and superparamagnetic clustering. Neural computation 16:1661–1687.

Rabang CF, Parthasarathy A, Venkataraman Y, Fisher ZL, Gardner SM, Bartlett EL (2012) A computational model of inferior colliculus responses to amplitude modulated sounds in young and aged rats. Frontiers in neural circuits 6:77.

bioRxiv preprint doi: https://doi.org/10.1101/2023.11.19.567743; this version posted November 21, 2023. The copyright holder for this preprint (which was not certified by peer review) is the author/funder, who has granted bioRxiv a license to display the preprint in perpetuity. It is made available under aCC-BY 4.0 International license.

Salvatier J, Wiecki TV, Fonnesbeck C (2016) Probabilistic programming in Python using PyMC3. PeerJ Computer Science 2:e55.

Simon H, Frisina RD, Walton JP (2004) Age reduces response latency of mouse inferior colliculus neurons to AM sounds. The Journal of the Acoustical Society of America 116:469–477.

Vehtari A, Gelman A, Gabry J (2017) Practical Bayesian model evaluation using leave-one-out cross-validation and WAIC. Stat Comput 27:1413–1432.

Wells J, Kao C, Mariappan K, Albea J, Jansen ED, Konrad P, Mahadevan-Jansen A (2005) Optical stimulation of neural tissue in vivo. Optics Letters 30:504.
